# Rare Case of a Chondrosarcoma of the Mandible in a Child

**DOI:** 10.1155/2013/837617

**Published:** 2013-11-18

**Authors:** Hendryk Vieweg, Jan Thiess Deichen, Fabian Scheer, Reimer Andresen, Roland Talanow

**Affiliations:** ^1^Institute of Diagnostic and Interventional Radiology/Neuroradiology, Westküstenklinikum Heide, Academic Teaching Hospital of the Universities of Kiel, Lübeck and Hamburg, 25746 Heide, Germany; ^2^Conradia Radiologische Praxen, 21029 Hamburg, Germany; ^3^InnoMed, LLC, Lake Tahoe, Stateline, NV 89449-4470, USA

## Abstract

Chondrosarcoma of the mandible is rare, especially in children. The available literature consists mostly of a few case reports which are partly integrated in small studies. Growing this small pool of literature is helpful in solidifying knowledge about this disease and facilitating appropriate treatment for children. Therefore, we present such a case in a 12-year-old boy, exhibit comprehensive and relevant information concerning this entity, and discuss our findings in the context of other publications.

## 1. Case Report

A 12-year-old boy was referred to our ambulant institution for an MRI examination of the right temporomandibular joint (TMJ). He reported to have received a hit on the right side of the face 6 months earlier and from that moment experienced intermittent cheek swelling and pain in combination with an incomplete ability to open the mouth. However, no diagnostic imaging was conducted since then. At the day of presentation, a distinct swelling around the parotid gland was observed. In his medical history, allergic asthma and neurodermitis were documented.

**Figure 1:**
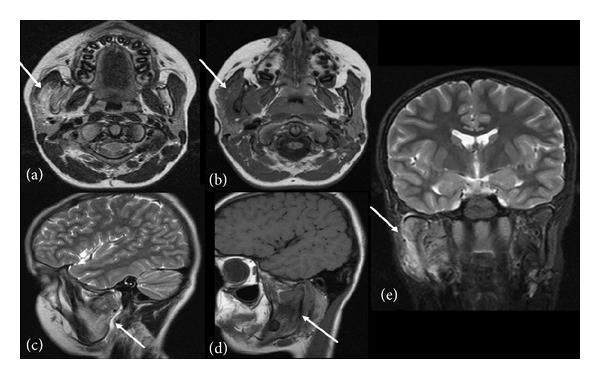
(a) T2-weighted axial 3 mm spin-echo sequences. (b) T1-weighted axial 3 mm spin-echo sequences. (c) T2-weighted sagittal 4 mm turbo spin-echo sequences. (d) T1-weighted sagittal 3 mm turbo spin-echo sequences. (e) T2-weighted coronal 4 mm turbo inversion recovery magnitude sequences. (a)–(e) MRI sequences reveal enlarged mandibular bone structures with a loss of regular bony cortex and a soft tissue mass infiltrating the temporal and masseter muscles.

The MRI scan revealed several findings. Extending from the middle third of the mandibular ramus to the mandibular head, the bony structures were enlarged with multifocal loss of bony cortex. Alterations in bone marrow signal intensity were encountered, T1-weighted signals were hypointense, and T2-weighted signals were isointense. The TMJ was not involved. A 33 × 28 × 25 mm measuring soft tissue mass was visible partly overlying most of the medial aspect of the mandible and infiltrating the temporal and masseter muscles (Figures 1(a)–1(e)). No suspicious lymph nodes were identified. The result of the MRI was not matching a posttraumatic status but was rather indicative of a primary malignant bone tumor.

In the following stationary setting, the patient was discussed within a multidisciplinary pediatric oncology tumor board. He received a PET-CT examination for staging reasons. The right mandible and its surrounding muscles showed a solitary FDG avid mass with no other evidence of disease. CT with oral and intravenous contrast confirmed subtotal destruction of the right mandibular head and both osteolytic and osteoblastic lesions in the mandibular ramus, matching the MRI results. The tumor was staged T2 N0 M0.

To classify the tumor surgical bone biopsy was performed to retrieve multiple osseous tissue samples. The pathology result and reference pathology were consistent with chondrosarcoma.

Wide resection was executed en bloc together with the biopsy scar and track followed by adequate reconstruction. 

## 2. Discussion

Chondrosarcoma is the second most frequently seen malignant bone tumor after osteosarcoma, accounting for about 20%. In adulthood, it is one of the most frequently occurring bone sarcomas. The tumors develop as primary entities or secondary to malignant transformation of benign enchondroma and osteochondroma. Syndromes of familial multiple enchondroma like Maffucci and Ollier as well as hereditary multiple exostoses (HME) are related to an increased risk of secondary chondrosarcoma, in the latter case affecting younger patients. 

The patient age ranges from 1 to 75, averaging between 30 and 60 years.

Generally, the tumor can arise in every region of the body where cartilage is present but it usually emerges from the axial skeleton, pelvic girdle, femur, humerus, vertebra, shoulder, sternum, or ribs [1]. 

About 90% of chondrosarcomas are classified central and are located intramedullary, 5% are extraskeletal myxoid, 2% each are mesenchymal and periosteal (juxtacortical), and 1% are clear cell.

There is no distinct gender predominance in conventional chondrosarcoma.

The mesenchymal type, which contains an extensive soft tissue component, appears in younger patients (second to fourth life decade) and is accompanied by a poorer prognosis [2]. 

The periosteal subtype is less aggressive and has a better prognosis [3]. 

Based on the mitotic index, cellularity and tumor size, low (80–90%) and higher grade malignancies were differentiated by Evans et al. in 1977, and this grading system is still most commonly used [4]. 

Low grade (G1) malignancy has an ample chondroid matrix with initial signs of hypercellularity, chondrocytes with an increased size, and sometimes slightly hyperchromatic, near normal nuclei forms. Slight-to-moderate atypia is seen but mitoses are quite rare.

Higher grade (G2/G3) malignancies have a less chondroid and more myxoid matrix, a higher degree of hypercellularity, a progressive increase in nuclear polymorphisms and enlargements, an increasing mitotic activity.

An additional immunohistochemical analysis of the biopsy can be helpful by checking the cellular proliferation markers Ki-67 and Cyclin B1, which are often increased.

Chondrosarcoma occurrence in the head and neck region is reported to be seen in only 5–12% of cases, involving mostly the maxilla, nasal septum and cavity, ethmoid bone, and sphenoid bone. Out of this minority of cases, the mandible accounts only for 10%, involving mostly the molar regions and mandibular symphysis. Chondrosarcoma in the ramus, head, or even TMJ is of absolute rareness [5].

However, especially the TMJ is a challenging location due to functional and aesthetic aspects like ingestion, speech, and facial expressions. 

Comparing the involvement of the maxilla and mandible, patients with the latter have been reported to be 4 years younger in average, explained by the fact that the mandible is more forming of the facial appearance and the maxilla offers more inner space to hide the tumor, causing a difference in the rapidity of diagnosis. The same explanation applies to the fact that chondrosarcoma of the mandibular symphysis seems to have the best prognosis [6].

Mostly pain and progressive, often painless, swelling are reported at doctor consultation. Those symptoms in the region of the TMJ, especially in combination with biting or chewing difficulties, are often misinterpreted as TMJ disorders. This can cause a significant delay in finding the correct diagnosis. If the tumor arises in the molar regions, loosening of the associated teeth and widening of the periodontal ligament spaces are important warning signals, especially for the dentist [7].

In our case, the patient reported a trauma as the starting point for the symptoms. This phenomenon has been documented in previous studies in a minority of patients and should therefore be an investigation parameter in possibly upcoming studies [6].

Diagnostic imaging is an important part of the diagnostic workup, especially to evaluate the size of the tumor, which has been reported to average at 4 cm, ranging from 1 to 12 cm, matching our maximum diameter of 3.3 cm [8].

The tumor site is another fundamental parameter because a solitary involvement of the medial cortical aspect of the mandible can cause a diagnostic delay due to missing symptoms while involvement of the lateral site leads to swelling, pain, and dysfunction. We could confirm this in our case in which the medial site was affected more so that the patient waited 6 months before seeing a doctor. 

Several radiological modalities are beneficial. Conventional radiography is often the first modality performed in the course of events and findings consist mostly of variable radiolucencies.

For further evaluation, MRI should be performed to have an overview of bone and soft tissue involvement and CT to assess the degree of bone destruction and the presence of calcifications. Those signs raise suspicion of a malignancy but are not pathognomonic for chondrosarcoma. Osteosarcoma can appear similar although calcifications are much more infrequent. Ultimately, biopsy has to be performed to establish the definite diagnosis.

In conventional chondrosarcoma, distant metastases are rare but sometimes seen in advanced or recurrent diseases. When they occur, they are usually detected in the lungs and bones, infrequently in lymph nodes, so that a neck dissection is not obligatory.

For the TMJ region, there are no reports about distant metastases in G1-chondrosarcoma, while they were seen in 10% of cases in G2-chondrosarcoma and 71% of cases in G3-chondrosarcoma [9].

Concerning therapy, due to a slow growth rate chondrosarcoma is little sensitive to radiochemotherapy, which is reserved for cases of unresectable tumor and incomplete resection margins and as an adjuvant therapy for patients with high grade tumors [10].

Radical surgery is the treatment of choice and surgical bone and soft tissue resection margins of 2-3 cm need to be tumor-free and are otherwise related to a poor prognosis.

After successful surgical therapy with inconspicuous resection margins, the 5-year survival rate is still only 50%, by reason of a tendency to recur locally uncontrollable. Prognosis is generally poorer in the head and neck region than in more frequently involved body parts.

Regarding pediatric patients with chondrosarcoma of the head and neck, we found one comparable case report of an 11-year-old girl with a chondrosarcoma of the TMJ. The main difference was that in our case the tumor did not actually reach the TMJ, affecting only the mandible [11].

In addition, we found a publication investigating primary chondrosarcoma of the head and neck in the pediatric age group, consisting of 14 patients of 18 years or of younger age with a mean age of 11.8 years. No genetic dispositions were detected. The tumors were mostly low grade and had a peak incidence in the maxillary sinus and mandible. The long-term prognosis was stated to be excellent [12].

Apart from this, pediatric cases were integrated in general chondrosarcoma studies, for instance, in reports from 1992 of 28 cases with 21.4% children or from 1995 of 634 patients with 2.2% children.

In both reports, the tumor in children was not more aggressive than in adults and there was no significant difference in the survival rate [8, 13].
